# Cerebral arterial architectonics and CFD simulation in mice with type 1 diabetes mellitus of different duration

**DOI:** 10.1038/s41598-021-83484-7

**Published:** 2021-02-17

**Authors:** Galina Yankova, Darya Tur, Daniil Parshin, Alexander Cherevko, Andrey Akulov

**Affiliations:** 1 Lavrentyev Institute of Hydrodynamics of the Siberian Branch of the Russian Academy of Sciences, Novosibirsk, Russia; 2 Institute of Cytology and Genetics of the Siberian Branch of the Russian Academy of Sciences, Novosibirsk, Russia

**Keywords:** Animal disease models, Statistics, Type 1 diabetes

## Abstract

Type 1 diabetes is a chronic autoimmune disease that affects tens of millions of people. Diabetes mellitus is one of the strongest factors in the development of cerebrovascular diseases. In this study we used NOD.CB17 Prkdcscid mice and the pharmacological model of type 1 diabetes mellitus of different duration to study changes in the cerebral vasculature. We used two combined approaches using magnetic resonance angiography both steady and transient CFD blood flow modeling. We identified the influence of type 1 diabetes on the architectonics and hemodynamics of the large blood vessels of the brain as the disease progresses. For the first time, we detected a statistically significant change in angioarchitectonics (the angles between the vessels of the circle of Willis, cross-sections areas of vessels) and hemodynamic (maximum blood flow rate, hydraulic resistance) in animals with diabetes duration of 2 months, that is manifested by the development of asymmetry of cerebral blood flow. The result shows the negative effect of diabetes on cerebral circulation as well as the practicability of CFD modeling. This may be of extensive interest, in pharmacological and preclinical studies.

## Introduction

More than 200 million people have been diagnosed with diabetes. About 10% of them are sick with type 1 diabetes. Type 1 diabetes mellitus is a widespread chronic autoimmune disease^1^. Long-term course of the disease leads to the development of associated illness^2^. Thus, diabetes mellitus is one of the strongest factors in the development of cerebrovascular diseases^3^. Multiple studies suggest that hyperglycemia can cause excess free fatty acids^4^, loss of endothelial nitric oxide^5,6^, prothrombotic state^7^, endothelial dysfunction^8,9^, abnormal release of endothelial vasoconstrictors^10^, vascular smooth muscle dysfunction^11^, oxidative stress^12,13^, which can lead to damage to blood vessels and the development of pathological blood flow in them. Vascular disorders can also occur in the cerebral vasculature. View of the above it is expected that with an increase in the diabetes duration, the changes in the cerebral vessels hemodynamics can potentially appear or intensify.

The cerebral vascular system has a complex structure. The basic component of the cerebral vasculature is a complex of large arteries consisting of carotid and vertebral arteries as well as the circle of Willis formed by anterior cerebral arteries, anterior communicating arteries, posterior cerebral arteries, posterior communicating arteries as well as middle cerebral artery^14^.

On the one hand, the structure of these vessels should be sufficiently conservative. On the other hand, it is natural to assume that with the course of the disease leading to changes in the functioning of the circulatory system and the need for its adaptation there may be a change in the geometry and hemodynamic regime of this arterial complex.

These arteries are the largest in the brain and therefore their architectonics is available for study by Magnetic Resonance Imaging (MRI) methods.

Blood vessel geometry data obtained by magnetic resonance angiography (MRA) can be used for subsequent CFD simulation. Since the hemodynamics study is associated with a complex flow geometry and complicated flow, application packages, ANSYS, COMSOL, OpenFOAM, etc.^15–17^, are often used to solve the problem of moving blood through the vessels.

In this work, the influence of type 1 diabetes lasting 1 and 2 months on the architectonics and hemodynamics of cerebral blood vessels was studied using NODSCID mice as model objects. We used a combined approach to obtain the actual configuration of the mice' cerebral vessels using MRI, then both steady and transient numerical analysis in the ANSYS CFX software and subsequent statistical analysis of geometric and hemodynamic characteristics using PLS-DA. The data supporting the conclusions of this article is included within the article in the Supplementary files.

## Materials and methods

### Object of study and experimental groups

All procedures were performed in accordance with the European Convention for the Protection of Vertebrate Animals used for Experimental and other Scientific Purposes. The experimental protocol was approved by the Bioethical Committees at the Institute of Cytology and Genetics of the Siberian Branch of the Russian Academy of Sciences. All animal experiments described below were in compliance with the ARRIVE (Animal Research: Reporting in Vivo Experiments) guidelines.

In this study we use NOD.CB17 Prkdc^scid^ mice (NOD SCID) from the Centre for Laboratory Animal Genetic Resources, Institute of Cytology and Genetics, Siberian Branch of the Russian Academy of Sciences (RFMEFI62119X0023). This line of mice is chosen because of their increased sensitivity in the pharmacological model of diabetes mellitus (streptozotocin injection).

At the beginning of the experiment animals of SPF (Specific Pathogen Free) status were at the age of 8 weeks. The diabetes group was injected intraperitoneally with streptozotocin (Sigma, USA) dissolved in 0.01 M citrate buffer at pH 4.2. A total of 150 mg streptozotocin/kg body weight was administered to induce diabetes. The control group was injected with the same volume of the citrate buffer.

Four groups of animals were formed: 1c—7 males; 1d—5 males; 2c—10 males; 2d—9 males. Here: 1, 2—the duration of the experiment in months, c—control, d—diabetes.

Initially, the number of animals in group 1d was 7. However, one animal died during the experiment (which corresponds to the acceptable percentage of mortality in the pharmacological model of diabetes). Another one from this group was excluded from the analysis due to the substandard quality of the MRA data.

To confirm the presence of type 1 diabetes in all animals used in the experiment a multiple measurement of blood glucose level (mmol/l) was carried out by electrochemical method^18^ using Diacont® glucometer (Diacont Ltd., Moscow, Russia) and individual test strips of the same company compatible with the glucometer. Measurements were carried out in animals before the experiment, 7 days after the introduction of streptozotocin and at the end of the experiment. Blood sampling was made from the tip of the animal's tail 4 h after removal of feed, so the blood was mixed type, taken on an empty stomach.

### MRA and cerebral vasculature reconstruction

MRA data were obtained on an ultrahigh-field tomograph with a magnetic field strength of 11.7 T (BioSpec 117/16USR, Bruker) with a volume RF coil for head of mouse MRA data. Images and the blood flow velocity in blood vessels of the mouse were recorded with a transmitter and receiver volume (T11232V3) 1H radiofrequency coil. The vessel’s architectonics was determined using 3D-TOF (Time of Flow) method^19^ with pulse sequence parameters Echo Time (TE) = 3.2 ms, and Repetition Time (TR) = 15 ms (three-dimensional image of the vessels with a field of view 2 cm × 2 cm × 2 cm and a matrix size of 256 × 256 × 128). The blood flow rate was determined in single slice (the common carotid arteries) using 2D phase-contrast magnetic resonance imaging method with pulse sequence parameters TE = 6 ms, TR = 20 ms (1- mm thick slice with a field of view of 2 cm × 2 cm and a matrix size of 256 × 256). The data obtained have a voxel size of 78 × 78 × 156 microns.

The small size of the mice cerebral vessels required an increase in image resolution. To do this, MRA data were interpolated to the matrix 512 * 512 * 256 in the Seg3D program^20^ using the Cubic (Catmull-Rom) interpolation method. This allowed us to obtain data of sufficient quality for CFD simulation without excessive use of interpolation.

**Figure 1:**
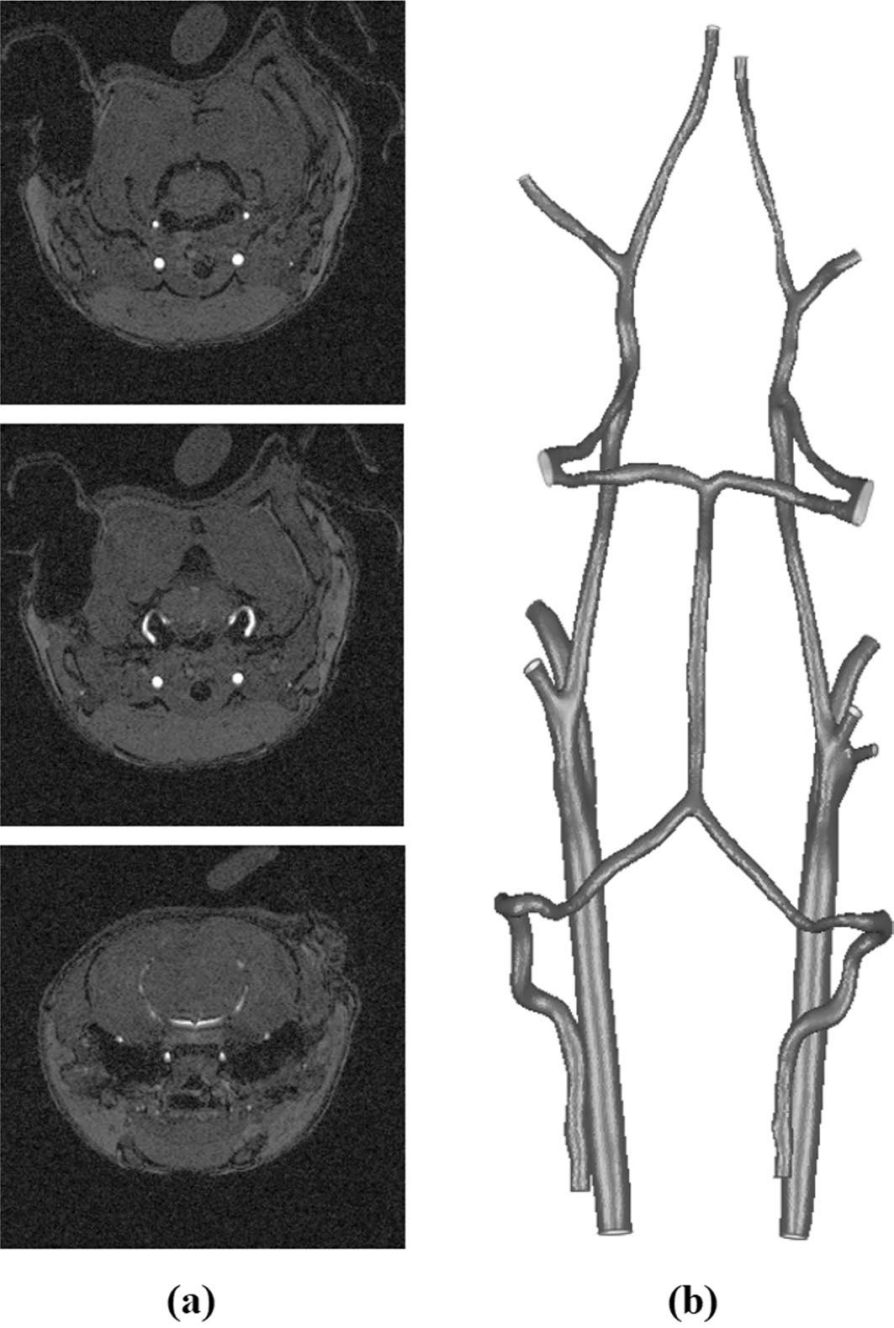
Reconstruction stages of the vascular network geometry for one of the animals: (**a**) MRI data, (**b**) vector image.

Non-fragmented three-dimensional models of vascular networks of all animals have been constructed using the software ITK-Snap for segmentation^21^. After segmentation, the images of the vessels were cleaned from noise, the cerebral arterial network was recognized, and the models smoothing was performed. Figure 1 shows the steps of reconstructing the vascular network geometry for one of the animals. The process of geometry construction was described in more detail in^22^.

### Angioarchitectonics study and CFD simulation

**Figure 2:**
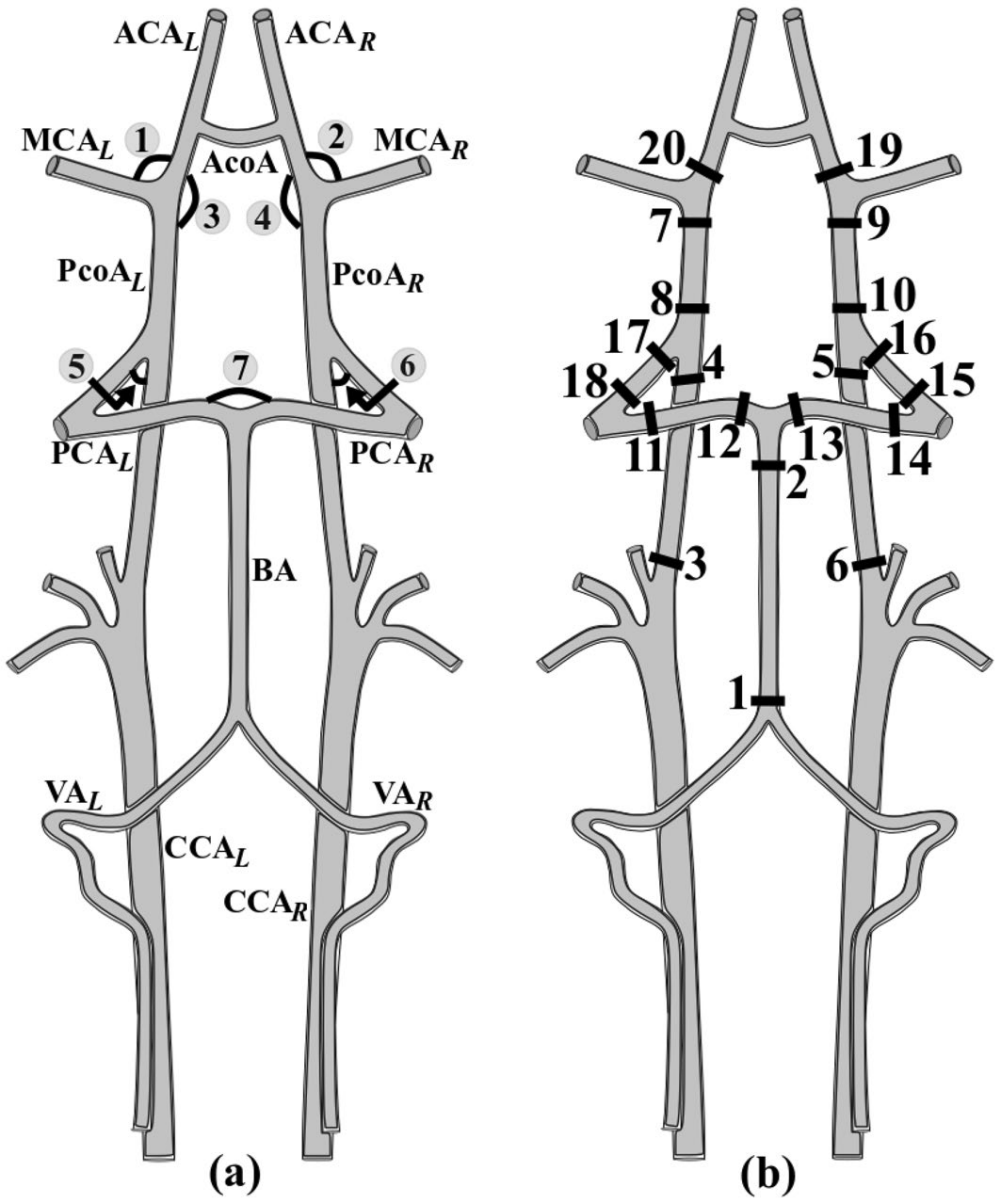
Anatomy of the mice circle of Willis (CoW). (**a**): The arteries are common carotid artery (CCA), vertebral artery (VA), basilar artery (BA), anterior cerebral artery (ACA), posterior cerebral artery (PCA), middle cerebral artery (MCA), posterior communicating artery (PcoA) and anterior communicating artery (AcoA). – L denotes left side, and − R—right side. 1 is the angle between ACA_L_ and MCA_L_, 2 is the angle between ACA_R_ and MCA_R_, 3—the angle between ACA_L_ and PcoA_L_, 4—the angle between ACA_R_ and PcoA_R_, 5—the angle between ICA_L_ and PcoA_L_, 6—the angle between ICA_R_ and PcoA_R_, 7—the angle between PCA_L_ and PCA_R_. (**b**): The cross-sectional planes.

At the obtained configurations of all mice, vascular networks geometrical parameters were measured. Namely, the angles $\alpha \left[ {{\text{degree}}} \right]{ }$ between the vessels of the circle of Willis, orthogonal cross-sections areas of vessels $S \left[ {mm^{2} } \right]$ in the selected position are shown in Fig. 2.

Software ANSYS CFX is used for numerical analysis of hemodynamics. Blood flow CFD calculations in the circle of Willis and adjoining arteries were carried out based on the obtained vascular geometry by MRA velocity data in the common carotid arteries. Verification of the reliability of the simulation by comparing high-quality experimental MRA data with the results of CFD modeling was carried out in our early works^23,24^.

In hemodynamic modeling, it is widely used both with and without flow pulsations^25,26^. In our case, the Womersley’s number is quite large and on the order of tens. Therefore, it is impossible to say in advance whether unsteady effects can be ignored. In this regard, we carry out both stationary and non-stationary CFD modeling.

The incompressible viscous liquid model is used for numerical modeling. Walls of blood vessels are considered to be the rigid and no-slip condition is used. Flow considered being laminar. Thus, the mathematical model of blood flow has the form:

$$\left\{ {\begin{array}{*{20}l} {div \vec{v} = 0,} \hfill \\ {\frac{{\partial \vec{v}}}{\partial t} = - \left( {\vec{v} \cdot \nabla } \right)\vec{v} - \frac{1}{\rho }\nabla p + \nu \Delta \vec{v}} \hfill \\ \end{array} } \right.$$

Here $\nu$—coefficient of kinematic viscosity, $\rho = const$—liquid density, $\vec{v}$—fluid velocity vector and $p$—pressure. The first equation expresses mass conservation law; the second equation is the law of conservation of impulse momentum in vector form.

For steady calculation at the inlets of vertebral arteries and common carotid arteries we set individual values of the average blood flow velocity obtained during the MRA measurements. These velocities are shown in the Supplementary (Table 5). At the outlets, we set a volumetric flow rate proportional to its cross-section area. Such outlet conditions make it possible to take into account the velocity profiles formed in the vessels near the outlet. For most outlets there is no need for long output sections of the vessels. For outlets 7 and 8, due to their proximity to vascular bifurcation, additional output Sects. 1 mm long were added. The correctness of this approach was validated by stationary calculations without lengthening the outputs.

For transient calculation at the inlets we used the same average velocity. The varying-time velocity pattern was used based on data from^27^. The outlets volumetric flow rates are proportional cross sectional areas and inlet velocity value in each timepoint. Lengthened outlets 7 and 8 are used. The transient calculation time = 0.137 s, which corresponds to one cardiac cycle, time step = 0.005 s.

**Figure 3:**
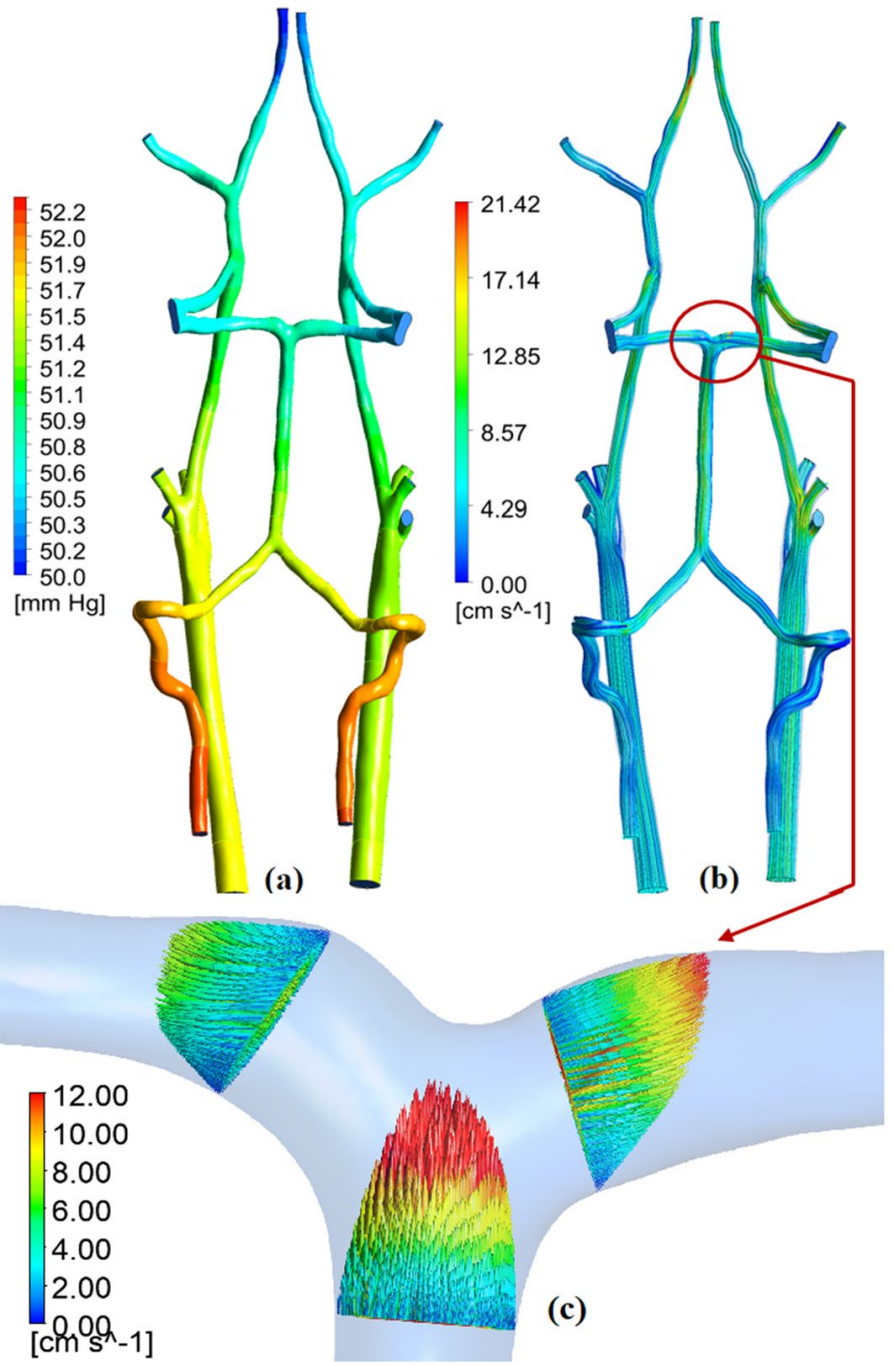
CFD simulation results for one of animals: (**a**) Pressure distribution on the wall of the circulatory system, (**b**) Stream lines of the velocity vector, (**c**) Velocity field on cross-sectional planes: №2, №12, №13.

Velocity and pressure values were obtained as a result of numerical calculations in the vasculature. In the transient case, their average values for the cardiac cycle are used. An example of pressure and velocity distribution within the circulatory system is shown in Fig. 3.

These CFD modeling results were used to obtain the following blood flow parameters:

Mass flow $Q_{i}$ $[mg \cdot s^{ - 1} ]$ though one of the sectional planes $\Omega_{i}$ in the Fig. 2 by definition is

$$ \mathop {\iint }\limits_{{\Omega_{i} }}^{ } \rho \left( {\vec{v} \cdot \vec{n}} \right)dS = Q_{i} , $$

Maximum blood flow velocity $\vec{v}_{i}^{max}$ $[cm \cdot s^{ - 1} ]$ through the section $\Omega_{i}$

$$ \mathop {\max }\limits_{{\Omega_{i} }} \vec{v} = \vec{v}_{i}^{max} , $$

Hydraulic resistance $R_{ij}$ $[mmHg \cdot s \cdot g^{ - 1} ]$ in the vascular network between sections $\Omega_{i}$ and ${\Omega }_{j}$

$$ \frac{{\Delta P_{ij} }}{{Q_{i} }} = R_{ij} $$

Here $S_{i}$—vessel cross-section area of section $\Omega_{i}$, $\vec{v}$—blood flow vector, $\vec{n}$—normal unit vector to the cross-section plane, $\rho$—blood density, $P_{i}$ – average total blood pressure in the vessel section, $\Delta P_{ij}$—pressure drop between sections.

Blood flow velocity characteristics, angioarchitectonics, and CFD simulation were carried out in accordance with the requirements of the blind study.

### Statistical data processing

As parameters for further statistical analysis were chosen: the angles between the vessels of the circle of Willis $\alpha$, cross-sections areas of vessels $S_{i}$, mass flow $Q_{i}$, maximum blood flow rate $\vec{v}_{i}^{max}$, the hydraulic resistance of the vascular network between sections $R_{ij}$ .

To establish the normal sample distribution of the estimated parameters, we used the Kolmogorov–Smirnov criterion. Further, the data are shown as *mean* ± *SE*. Comparison between groups was also made using parametric statistics methods—Student's t-test (p-value less than or equal to 0.05 is considered statistically significant).

The number of initial variables was reduced with partial least-squares discriminant analysis (PLS-DA), which is widely used in biological and medical research because it allows maximization of the separation between groups of observation^28^. The PLS-DA builds a linear regression model by projecting the variables to a new space, with Y as a categorical variable^29^. Additionally, PLS-DA model was tested using a stepwise variable elimination procedure. Then two-way ANOVA (on pathology and disease duration) was performed, with Y values as the dependent variable, in order to assess the distribution of experimental groups in the space of Y axes.

## Results

Data on glucose levels for all animal groups are given in Supplementary (Table 1).

Regarding the MRA data, in group 1d the blood flow rate in both the right and left common carotid arteries is significantly lower than in group 1c. In addition, there is no significant asymmetry of blood flow in both groups. There are no differences in the blood flow rate in the left common carotid artery in animals of group 2d compared to group 2c, but there is a significant decrease in blood flow in the right common carotid artery. At the same time, a significant asymmetry of blood flow is observed only in group 2d. Data about all four groups are shown in Supplementary (Table 2).

Generally the analysis showed the absence of the T1Dm effect for 1 month on the geometric and hemodynamical characteristics of the investigated vessels, except for one of the 73 parameters: in steady calculation (the maximum velocity in cross Sect. 22—group 1c: 12.67 ± 1.471; group 1d: 8.33 ± 0.766; p = 0.04) and in transient calculation (the maximum velocity in cross Sect. 22—group 1c: 12.06 ± 1.326; group 1d: 8.13 ± 0.666; p = 0.04), see Supplementary (Tables 3 and 4).

At the same time animals group 2d have significant differences in the geometric characteristics in comparison with the group 2c. Significant changes in cross-sectional areas in the cervical segment C1 were found: Sect. 3 area of left internal carotid artery (group 2c: 0.12 ± 0.009; group 2d: 0.09 ± 0.005; p = 0.03) and Sect. 6 area of right internal carotid artery (group 2c: 0.10 ± 0.008; group 2d: 0.07 ± 0.004; p = 0.01). Also difference in the base of the Sect. 1 area basilar artery (group 2c: 0.06 ± 0.006; group 2d: 0.10 ± 0.016; p = 0.01) was detected. Also, significant difference was found in the angle between right ACA and MCA (group 2c: 67.60 ± 2.663; group 2d: 60.22 ± 2.783; p = 0.04).

In steady calculation a significant difference in hemodynamics between groups 2c and 2d is observed in: hydraulic resistance between planes 5 and 6 (group 2c: 57.18 ± 4.679; group 2d: 97.64 ± 15.565; p = 0.02); the maximum velocity in cross Sect. 1 (group 2c: 15.22 ± 0.919; group 2d: 9.98 ± 1.091; p = 0.02), Sect. 15 (group 2c: 25.96 ± 5.461; group 2d: 9.49 ± 1.406; p = 0.01). Also there were several of parameters closely approaches the brink of significance: mass flow rate between Sect. 15 and 16 (group 2c: 4.70 ± 0.001; group 2d: 2.60 ± 0.001; p = 0.07); the maximum velocity in cross Sect. 13 (group 2c: 9.45 ± 1.230; group 2d: 17.89 ± 4.230; p = 0.06), Sect. 16 (group 2c: 15.55 ± 2.790; group 2d: 9.05 ± 1.854; p = 0.08).

Transient calculation shows corresponding results. Hydraulic resistance between planes 5 and 6 (group 2c: 60.95 ± 5.893; group 2d: 99.24 ± 14.945; p = 0.02); the maximum velocity in cross Sect. 1 (group 2c: 14.22 ± 0.649; group 2d: 9.59 ± 1.038; p = 0.02), Sect. 15 (group 2c: 24.77 ± 4.851; group 2d: 9.02 ± 1.396; p = 0.01). Also there were several of parameters are closely approaches the brink of significance: mass flow rate between Sect. 15 and 16 (group 2c: 4.52 ± 0.001; group 2d: 2.45 ± 0.0004; p = 0.052); the maximum velocity in cross Sect. 13 (group 2c: 9.04 ± 1.129; group 2d: 16.79 ± 3.880; p = 0.06), Sect. 16 (group 2c: 15.01 ± 2.456; group 2d: 8.84 ± 1.782; p = 0.06). Comprehensive data set included in Supplementary (Tables 3 and 4).

**Figure 4:**
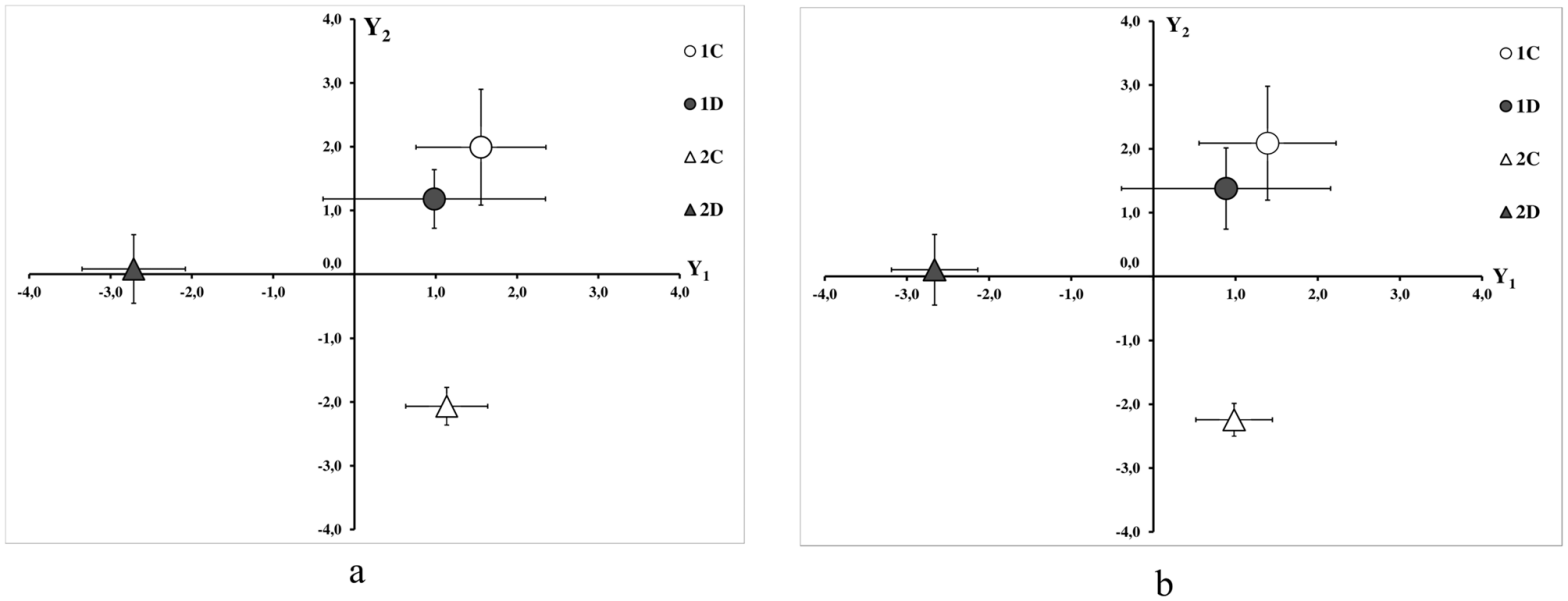
PLS-DA results: (**a**) steady calculation, (**b**) transient calculation. Groups of animals: 1, 2—the duration of the experiment in months, (**c**)—control, (**d**)—diabetes. Values are presented as mean $\pm$ SE.

Partial least square discriminant analysis (PLS-DA) was performed on the basis of the obtained vessel geometry data and CFD simulation results. The similarity of the results of steady and transient calculations is reflected in the similar results of PLS-DA (Fig. 4). According to PLS-DA data no significant differences in the Y1 and Y2 axes were found for the group 1c and 1d animals. Whereas animals group 2c and 2d are significantly different along the Y1 axis. When comparing animals 1c vs. 2c and 1d vs. 2d there are significant differences along the Y2 axis. Thus, the Y1 axis is associated with disease progression over time, and the Y2 axis is associated with animal age. These findings are supported by two- way analysis of variance (ANOVA). The steady case analysis showed that both the duration of the experiment (F_1,28_ = 7.08, p = 0.01) and the disease (F_1,28_ = 8.18, p = 0.01) have a significant influence on the Y1 axis. Only the duration of the experiment has a significant effect on the Y2 axis (F_1,28_ = 18.85, p < 0.01). The transient case analysis showed that both the duration of the experiment (F_1,28_ = 7.55, p = 0.01) and the disease (F_1,28_ = 8.32, p = 0.01) have a significant influence on the Y1 axis. Only the duration of the experiment has a significant effect on the Y2 axis (F_1,28_ = 22.01, p < 0.01).

## Discussion

In a study on a model object—the NODSCID mice, we used two combined approach using MRA together with CFD steady or transient blood flow modeling. We identified the influence of type 1 diabetes on the architectonics and hemodynamics of the major blood vessels of the brain as the disease progresses. It was confirmed statistically, in particular by PLS-DA analysis. Steady and transient modeling showed similar results.

The geometry changing of large vessels is possible in a number of pathologies^30,31^. At the same time, if these pathologies are not sudden in nature, for example, a stroke or trauma, then such changes are formed gradually^32^. Diabetes mellitus as a chronic disease has a long-term effect on blood vessels with the first time microvascular and then macrovascular changes^33^.

The vascular network structure of the brain has the ability of significant flow compensation^34^. An important role in the implementation of such compensation is assigned to the circle of Willis. Its configuration, although conservative, is still capable of significant variations^35^. No less important factor for the compensation can be a change in the lumen of blood vessels. According to the results of angiography, we did not observe acute occlusion in animals which could lead to a brain infarction. But we detected a decrease in the lumen of the internal carotid arteries only in animals with disease duration of 2 months. The reason that led to decrease in the lumen of vessels was not examined in this work. Perhaps one of the reasons that led to a decrease in the lumen of vessels could be atherosclerotic vascular changes caused by prolonged hyperlipidemia^36,37^. Metabolic disorder can also lead to the development of hypertension^38^. According to the European Society of Cardiology-EASC and the European Association for the Study of diabetes-EASD in Europe, the frequency of detection of arterial hypertension in type 1 diabetes mellitus is 10–30%, and in type 2 diabetes mellitus, this frequency reaches 70–80%. Since hypertension can affect blood flow characteristics, we set special boundary conditions when performing CFD calculations: the values of fluxes obtained from high-field MRA data individually for each animal were set as boundary conditions. We detected a decrease in blood flow velocity in the internal carotid arteries both for animals with diabetes duration 1 and 2 months, but only in animals with a duration of 2 months, this led to a significant change in hemodynamic characteristics. Apparently, such changes were the result of a combination of a decrease in blood flow velocity and a narrowing of the vessel lumen, which was observed only in animals with diabetes duration of 2 months. Since the change in blood vessels occurs gradually, the duration of diabetes for 2 months is sufficient for the influence of factors such as the loss of endothelial nitric oxide, endothelial dysfunction, oxidative stress and abnormal release of endothelial vasoconstrictors to become significant.

The decrease in the blood flow rate in animals with diabetes can be explained by changes in the volume characteristics of the left ventricular ejection fraction of the heart. Systolic heart failure is a common comorbidity in diabetes mellitus while the pathogenesis, in this case, may be due to myocardial nutritional disorders^39^. Systolic heart failure with high glucose and no treatment can occur in a short time^40^. Another result is a lower blood flow velocity tendency in the right internal carotid artery compared with the left one, regardless of the animal's group. This result is a physiological norm caused by anatomical features of the blood supply to the common carotid arteries from the aortic arch basin^41^. Note that, only in one (a diabetes duration of 2 months) of the four groups the differences in blood flow velocity between the internal carotid arteries were statistically significant. In this group, the blood flow rate in the right internal carotid artery was significantly lower than in the left one. Some diseases at a certain stage of development can lead to the formation of temporary or stable fluctuating asymmetry^42^. The asymmetry of a wide range of organism’s characteristics may indicate disorders^43^. What has been demonstrated recently, including in relation to the disease of diabetes mellitus^44,45^ and diabetes-related vascular disorders^46^. In a recent paper, an approach to analyzing the fluctuating asymmetry of fingerprints was used as an element of a prognostic tool for detecting diabetes mellitus type 1 and 2^47^. This can be based on epigenetic changes on the platform of emerging genetic noise when asymmetric gene expression occurs in the left and right parts of the body. Thus, we can assume that the genetic manifestation of asymmetric changes in hemodynamics is observed only in a group of animals with duration of diabetes of 2 months. This is manifested in an increase in hydraulic resistance between Sects. 5 and 6, a decrease in the maximum velocity of blood flow in Sect. 15, as well as several of parameters are closely approaches the brink of significance (See Fig. 2). It is worth noting that all these changes are located on the right side and belong to the posterior portion of the circle of Willis. In the study of Raghavendra and co-authors^48^ carried out on the posthumous material of people it is noted that against the background of the large variability of the circle of Willis organization, the posterior communicating artery is the most variable. Unfortunately, there is no information about what diseases people had during their lifetime, and the study was carried out on one ethnic group, although researchers point to similarities with data obtained by other researchers^49,50^. An interesting fact is that according to our data, no hemodynamic changes have been detected on the downstream right side while there was a change in the angle between the ACA and MCA. Is it related to the fact that the normalization of hemodynamics occurs on the same segment where the divergence angle of the middle cerebral artery changes? In a number of papers, it is noted that with an increase in blood pressure on the vessel walls, it is possible to change it geometry as a result of the change of the expression level of the genes responsible for the endothelium growth factor, cell proliferation change and migration^51,52^. In our case, it is possible that this change in angle has a compensatory nature since a change in the divergence angle has a significant effect on the hydraulic resistance^53^.

The work does not concern a number of important issues, the consideration of which would be interesting and which can be considered in future studies. Our research was focused on modeling the situation when endogenous insulin ceases to be produced at a young age and insulin therapy is not provided for a long time or is insufficient, which occurs, for example, in patients if the disease was not detected in time. This study did not concern the change in angioarchitectonics in type 1 diabetes on the background of insulin therapy that requires another one. We also do not consider the issue of type 2 diabetes mellitus, and, although it is also accompanied by hyperglycemia, nevertheless arises in an older age, has another pathogenesis and therefore requires another study. Our model is implemented in young animals, and therefore not quite correct to study vascular changes in type 2 diabetes mellitus.

## Conclusion

Thus, in our work, it is demonstrated that more strongly statistically significant differences in angioarchitectonics and hemodynamics are observed in animals with diabetes duration of 2 months, than in 1 month one. This was valid for both steady and transient simulations. The result shows the negative effect of diabetes on cerebral circulation as well as practicability of CFD modeling. This may be of extensive interest, for example, for pharmacological and preclinical studies. To sum up, with an increase in the duration of the disease, type 1 diabetes begins to influence the angioarchitectonics and hemodynamics of the large cerebral vessels of mice.

## Supplementary Information

Supplementary Information.
