# Supplementary material for: Cerebral arterial architectonics and CFD simulation in mice with type 1 diabetes mellitus of different duration
Source: Sci Rep. 2021 Feb 17;11:3969. doi: 10.1038/s41598-021-83484-7 (PMC7889636; doi:10.1038/s41598-021-83484-7)
Supplement: Supplementary file 1 — Supplementary Information. [file 41598_2021_83484_MOESM1_ESM.docx]

Cerebral arterial architectonics and CFD simulation in mice with type 1 diabetes mellitus of different duration

Galina Yankova*^1, 3^, Darya Tur^2^*,* ^3^, Daniil Parshin^1^*,* ^3^, Alexander Cherevko^1^*,* ^3^ , Andrey Akulov^2^*,*^3^

^1^ Lavrentyev Institute of Hydrodynamics of the Siberian Branch of the Russian Academy of Sciences, Novosibirsk, Russia

^2^ Institute of Cytology and Genetics of the Siberian Branch of the Russian Academy of Sciences, Novosibirsk, Russia

^3^ Novosibirsk State University, Novosibirsk, Russia

*** Correspondence:**Galina Yankova: [galinayankova2703@gmail.com](mailto:galinayankova2703@gmail.com)

## Supplementary Material

**Table 1. Blood glucose concentration**$[mmol/l]$

| **Groups** | **Blood glucose concentration,** $[mmol/l]$ | | |
| --- | --- | --- | --- |
|  | **at the beginning of**  **the experiment** | **in 7 days** | **at the end of the**  **experiment** |
| 1c | 6.6±0.5 | 5.7±0.5 | 6.8±0.5 |
| 1d | 6.5±1.1 | 16.5±1.1 | 29.0±4.5 |
| 2c | 6.3±1.6 | 6.2±0.6 | 6.6±0.9 |
| 2d | 6.2±0.6 | 19.9±8.2 | 22.9±8.9 |

**Table 2. Blood flow rates in the common carotid arteries**$[cm\cdot s^{-1}]$

|  | **Group 1c** | **Group 1d** | **1d vs. 1c, p** |
| --- | --- | --- | --- |
| **CCAL** | 8.18±0.692 | 12.03±0.788 | 0.004 |
| **CCAR** | 7.61±0.924 | 11.19±0.995 | 0.024 |
| **CCAL vs. CCAR, p** | 0.273 | 0.391 |  |
|  | **Group 2c** | **Group 2d** | **2d vs. 2c, p** |
| **CCAL** | 9.70±1.125 | 10.23±0.857 | 0.719 |
| **CCAR** | 7.76±0.544 | 9.92±0.837 | 0.039 |
| **CCAL vs. CCAR, p** | 0.016 | 0.596 |  |

In Table 3 we denote $R$ — vascular hydraulic resistance, $[mmHg s g^{-1}]$; $S$ — vascular cross-section area, $[mm^{2}]$; $Q$ — blood volume flow rate, $[mg s^{-1}]$; $v^{max}$ — maximum blood velocity, $[cm s^{-1}]$.

Indices denote: $R$ – right vasculature, $L$ – left vasculature, $M$ – medium vasculature. Number indices denote numbers of cross-section planes.

The data are shown as *mean* ± *SE*. Statistically significant values ($p$) are less than 0.05.

**Table 3. Comparison of mouse groups in the steady calculation**

|  | **Group 1c** | **Group 1d** |  | **Group 2c** | **Group 2d** |  |
| --- | --- | --- | --- | --- | --- | --- |
| **Parameter** | **mean ± S.E.** | **mean ± S.E.** | **p** | **mean ± S.E.** | **mean ± S.E.** | **p** |
| (ACA MCA) /L | 66.43 ± 2.768 | 67.20 ± 2.782 | 0.89 | 66.70 ± 1.416 | 70.78 ± 2.272 | 0.41 |
| (ACA MCA) /R | 71.29 ± 7.861 | 64.00 ± 2.354 | 0.49 | 67.60 ± 1.218 | 59.50 ± 2.783 | **0.04** |
| (ACA PcoA) /L | 163.29 ± 1.190 | 161.20 ± 4.572 | 0.46 | 162.40 ± 3.609 | 163.78 ± 1.489 | 0.51 |
| (ACA PcoA) /R | 155.57 ± 1.556 | 158.20 ± 5.580 | 0.65 | 161.80 ± 4.091 | 161.67 ± 1.772 | 0.95 |
| (ICA PcoA) /L | 54.57 ± 1.888 | 57.00 ± 4.593 | 0.37 | 56.20 ± 2.663 | 55.56 ± 1.701 | 0.79 |
| (ICA PcoA) /R | 53.14 ± 1.580 | 54.00 ± 1.483 | 0.73 | 54.60 ± 1.679 | 58.56 ± 1.482 | 0.10 |
| PCA /L PCA /R | 123.86 ± 5.680 | 132.00 ± 1.761 | 0.32 | 114.00 ± 1.720 | 122.56 ± 5.580 | 0.21 |
| $R_{1,2}$ /M | 140.76 ± 30.486 | 125.98 ± 42.362 | 0.78 | 135.88 ± 17.411 | 137.50 ± 31.339 | 0.96 |
| $R_{3,4}$ /L | 50.90 ± 8.119 | 72.11 ± 18.239 | 0.27 | 51.12 ± 8.016 | 66.57 ± 6.094 | 0.15 |
| $R_{5,6}$ /R | 57.65 ± 5.585 | 87.44 ± 30.877 | 0.29 | 57.18 ± 4.679 | 97.64 ± 15.565 | **0.02** |
| $R_{7,8}$ /L | 29.36 ± 6.984 | 35.87 ± 13.476 | 0.65 | 24.62 ± 3.349 | 29.35 ± 3.226 | 0.33 |
| $R_{9,10}$ /R | 31.40 ± 6.599 | 47.21 ± 23.954 | 0.48 | 34.69 ± 3.730 | 38.44 ± 5.896 | 0.59 |
| $R_{11,12}$/L | 129.47 ± 27.525 | 202.04 ± 77.181 | 0.34 | 114.03 ± 19.105 | 189.47 ± 77.600 | 0.34 |
| $R_{13,14}$ /R | 189.57 ± 96.984 | 112.60 ± 57.880 | 0.55 | 194.24 ± 71.783 | 100.72 ± 34.152 | 0.27 |
| $R_{15,16}$ /R | 124.42 ± 26.572 | 165.80 ± 108.637 | 0.67 | 194.44 ± 57.741 | 98.08 ± 16.885 | 0.15 |
| $R_{17,18}$ /L | 108.75 ± 31.488 | 173.93 ± 56.397 | 0.30 | 85.53 ± 14.623 | 88.08 ± 17.127 | 0.91 |
| $R_{19,20}$ /R | 160.12 ± 31.221 | 163.90 ± 22.648 | 0.93 | 170.38 ± 49.035 | 257.12 ± 50.590 | 0.24 |
| $R_{20,21}$ /L | 126.45 ± 24.668 | 134.78 ± 29.285 | 0.83 | 219.40 ± 39.776 | 164.92 ± 27.526 | 0.29 |
| $S_{1}$ /M | 0.07 ± 0.009 | 0.07 ± 0.010 | 0.71 | 0.06 ± 0.006 | 0.11 ± 0.016 | **0.01** |
| $S_{2}$ /M | 0.06 ± 0.008 | 0.07 ± 0.008 | 0.62 | 0.06 ± 0.009 | 0.07 ± 0.012 | 0.45 |
| $S_{3}$ /L | 0.10 ± 0.004 | 0.09 ± 0.009 | 0.34 | 0.12 ± 0.009 | 0.09 ± 0.005 | **0.03** |
| $S_{4}$ /L | 0.12 ± 0.010 | 0.09 ± 0.014 | 0.14 | 0.11 ± 0.010 | 0.10 ± 0.006 | 0.16 |
| $S_{5}$ /R | 0.10 ± 0.008 | 0.09 ± 0.015 | 0.58 | 0.10 ± 0.009 | 0.08 ± 0.006 | 0.09 |
| $S_{6}$ /R | 0.08 ± 0.004 | 0.07 ± 0.006 | 0.22 | 0.10 ± 0.008 | 0.07 ± 0.004 | **0.01** |
| $S_{7}$ /L | 0.06 ± 0.006 | 0.06 ± 0.010 | 0.84 | 0.07 ± 0.007 | 0.07 ± 0.007 | 0.70 |
| $S_{8}$ /L | 0.07 ± 0.008 | 0.07 ± 0.010 | 0.92 | 0.07 ± 0.005 | 0.08 ± 0.005 | 0.76 |
| $S_{9}$ /R | 0.06 ± 0.007 | 0.06 ± 0.011 | 0.96 | 0.05 ± 0.003 | 0.06 ± 0.008 | 0.64 |
| $S_{10}$ /R | 0.07 ± 0.007 | 0.06 ± 0.009 | 0.62 | 0.06 ± 0.003 | 0.07 ± 0.007 | 0.86 |
| $S_{11}$ /L | 0.05 ± 0.007 | 0.04 ± 0.007 | 0.59 | 0.05 ± 0.005 | 0.04 ± 0.006 | 0.34 |
| $S_{12}$ /L | 0.05 ± 0.007 | 0.06 ± 0.011 | 0.58 | 0.05 ± 0.007 | 0.05 ± 0.009 | 0.83 |
| $S_{13}$ /R | 0.08 ± 0.006 | 0.07 ± 0.012 | 0.68 | 0.05 ± 0.006 | 0.04 ± 0.005 | 0.25 |
| $S_{14}$ /R | 0.03 ± 0.004 | 0.03 ± 0.005 | 0.87 | 0.04 ± 0.006 | 0.04 ± 0.003 | 0.69 |
| $S_{15}$ /R | 0.05 ± 0.005 | 0.05 ± 0.015 | 0.93 | 0.04 ± 0.006 | 0.05 ± 0.006 | 0.20 |
| $S_{16}$ /R | 0.05 ± 0.006 | 0.05 ± 0.010 | 0.56 | 0.05 ± 0.004 | 0.05 ± 0.004 | 0.92 |
| $S_{17}$ /L | 0.06 ± 0.003 | 0.06 ± 0.008 | 0.92 | 0.04 ± 0.004 | 0.05 ± 0.008 | 0.27 |
| $S_{18}$ /L | 0.05 ± 0.007 | 0.06 ± 0.011 | 0.47 | 0.04 ± 0.005 | 0.05 ± 0.005 | 0.29 |
| $S_{19}$ /R | 0.05 ± 0.010 | 0.06 ± 0.007 | 0.48 | 0.04 ± 0.004 | 0.04 ± 0.006 | 0.90 |
| $S_{20}$ /L | 0.06 ± 0.012 | 0.06 ± 0.008 | 0.94 | 0.06 ± 0.008 | 0.06 ± 0.005 | 0.93 |
| $S_{21}$ /L | 0.06 ± 0.014 | 0.05 ± 0.003 | 0.32 | 0.04 ± 0.006 | 0.04 ± 0.005 | 0.38 |
| $S_{22}$ /R | 0.04 ± 0.006 | 0.04 ± 0.003 | 0.89 | 0.04 ± 0.004 | 0.05 ± 0.006 | 0.26 |
| $Q_{1,2}$ /M | 6.01 ± 0.001 | 6.74 ± 0.001 | 0.49 | 5.84 ± 0.001 | 5.45 ± 0.000 | 0.51 |
| $Q_{3,4}$ /L | 9.49 ± 0.002 | 6.59 ± 0.002 | 0.26 | 9.54 ± 0.001 | 9.87 ± 0.002 | 0.89 |
| $Q_{5,6}$ /R | 7.78 ± 0.002 | 7.78 ± 0.002 | 1.00 | 7.81 ± 0.002 | 5.08 ± 0.001 | 0.14 |
| $Q_{7,8}$ /L | 6.97 ± 0.002 | 4.02 ± 0.004 | 0.30 | 4.54 ± 0.001 | 4.98 ± 0.001 | 0.71 |
| $Q_{9,10}$ /R | 4.27 ± 0.001 | 4.17 ± 0.004 | 0.94 | 3.25 ± 0.001 | 3.37 ± 0.001 | 0.88 |
| $Q_{11,12}$/L | 3.24 ± 0.001 | 4.70 ± 0.002 | 0.52 | 3.75 ± 0.001 | 2.58 ± 0.001 | 0.29 |
| $Q_{13,14}$ /R | 3.79 ± 0.001 | 4.58 ± 0.001 | 0.67 | 3.07 ± 0.001 | 4.46 ± 0.001 | 0.33 |
| $Q_{15,16}$ /R | 3.71 ± 0.001 | 4.06 ± 0.001 | 0.82 | 4.70 ± 0.001 | 2.60 ± 0.001 | **0.07** |
| $Q_{17,18}$ /L | 5.25 ± 0.002 | 4.90 ± 0.001 | 0.88 | 5.07 ± 0.001 | 5.09 ± 0.001 | 0.99 |
| $Q_{19,20}$ /R | 3.14 ± 0.001 | 2.30 ± 0.002 | 0.46 | 1.72 ± 0.001 | 1.63 ± 0.001 | 0.88 |
| $Q_{20,21}$ /L | 4.56 ± 0.002 | 2.13 ± 0.002 | 0.31 | 2.82 ± 0.001 | 3.24 ± 0.001 | 0.62 |
| $v_{1}^{max}$ /M | 15.13 ± 2.120 | 15.61 ± 2.597 | 0.89 | 15.22 ± 0.919 | 9.98 ± 1.091 | **0.002** |
| $v_{2}^{max}$ /M | 17.59 ± 1.920 | 18.77 ± 3.248 | 0.75 | 21.00 ± 1.772 | 18.67 ± 2.224 | 0.42 |
| $v_{3}^{max}$ /L | 15.74 ± 1.935 | 12.40 ± 2.452 | 0.30 | 13.12 ± 1.183 | 15.94 ± 2.070 | 0.24 |
| $v_{4}^{max}$ /L | 17.08 ± 2.965 | 13.90 ± 4.425 | 0.55 | 15.19 ± 1.701 | 17.12 ± 2.724 | 0.55 |
| $v_{5}^{max}$ /R | 13.67 ± 2.717 | 14.52 ± 2.168 | 0.82 | 14.58 ± 2.982 | 11.45 ± 2.367 | 0.43 |
| $v_{6}^{max}$ /R | 14.71 ± 2.076 | 14.85 ± 2.173 | 0.96 | 12.98 ± 1.841 | 12.00 ± 2.038 | 0.73 |
| $v_{7}^{max}$ /L | 17.57 ± 4.599 | 12.52 ± 2.362 | 0.41 | 10.93 ± 1.438 | 11.70 ± 0.986 | 0.67 |
| $v_{8}^{max}$ /L | 15.80 ± 3.418 | 12.33 ± 2.076 | 0.45 | 10.45 ± 1.061 | 11.60 ± 1.504 | 0.53 |
| $v_{9}^{max}$ /R | 13.63 ± 3.777 | 13.43 ± 2.667 | 0.97 | 10.44 ± 1.978 | 10.79 ± 1.401 | 0.89 |
| $v_{10}^{max}$ /R | 12.33 ± 3.385 | 12.75 ± 2.068 | 0.93 | 9.11 ± 1.684 | 9.01 ± 0.901 | 0.96 |
| $v_{11}^{max}$ /L | 16.45 ± 5.278 | 22.99 ± 10.020 | 0.55 | 15.89 ± 4.568 | 10.74 ± 2.346 | 0.35 |
| $v_{12}^{max}$ /L | 13.72 ± 4.216 | 14.57 ± 7.546 | 0.92 | 16.57 ± 5.449 | 9.28 ± 2.118 | 0.25 |
| $v_{13}^{max}$ /R | 11.64 ± 2.917 | 14.23 ± 3.973 | 0.60 | 9.45 ± 1.230 | 17.89 ± 4.230 | **0.06** |
| $v_{14}^{max}$ /R | 15.66 ± 3.241 | 22.70 ± 9.943 | 0.46 | 14.49 ± 3.261 | 19.68 ± 4.866 | 0.38 |
| $v_{15}^{max}$ /R | 17.79 ± 5.622 | 26.88 ± 15.035 | 0.54 | 25.97 ± 5.461 | 9.49 ± 1.406 | **0.01** |
| $v_{16}^{max}$ /R | 12.85 ± 3.432 | 18.38 ± 6.822 | 0.45 | 15.55 ± 2.790 | 9.05 ± 1.854 | **0.08** |
| $v_{17}^{max}$ /L | 14.73 ± 3.955 | 16.26 ± 3.605 | 0.79 | 19.83 ± 4.908 | 15.69 ± 3.206 | 0.50 |
| $v_{18}^{max}$/L | 27.94 ± 11.337 | 24.83 ± 4.838 | 0.83 | 22.44 ± 5.632 | 18.02 ± 3.264 | 0.52 |
| $v_{19}^{max}$ /R | 12.09 ± 3.141 | 7.59 ± 0.895 | 0.27 | 7.60 ± 1.967 | 8.13 ± 1.527 | 0.84 |
| $v_{20}^{max}$ /L | 10.94 ± 2.981 | 7.49 ± 1.397 | 0.38 | 8.27 ± 1.275 | 9.33 ± 1.182 | 0.55 |
| $v_{21}^{max}$ /L | 13.97 ± 3.080 | 8.04 ± 0.470 | 0.14 | 14.18 ± 3.333 | 8.05 ± 0.786 | 0.11 |
| $v_{22}^{max}$/R | 12.67 ± 1.471 | 8.33 ± 0.766 | **0.04** | 7.89 ± 1.903 | 11.99 ± 1.378 | 0.10 |

**Table 4. Comparison of mouse groups in the transient calculation**

|  | **Group 1c** | **Group 1d** |  | **Group 2c** | **Group 2d** |  |
| --- | --- | --- | --- | --- | --- | --- |
| **Parameter** | **mean ± S.E.** | **mean ± S.E.** | **p** | **mean ± S.E.** | **mean ± S.E.** | **p** |
| (ACA MCA) /L | 66.43 ± 2.768 | 67.20 ± 5.580 | 0.89 | 66.70 ± 4.091 | 70.78 ± 2.272 | 0.41 |
| (ACA MCA) /R | 71.29 ± 7.861 | 64.00 ± 4.593 | 0.49 | 67.60 ± 2.663 | 59.50 ± 2.783 | **0.04** |
| (ACA PcoA) /L | 163.29 ± 1.190 | 161.20 ± 2.782 | 0.46 | 162.40 ± 1.416 | 163.78 ± 1.489 | 0.51 |
| (ACA PcoA) /R | 155.57 ± 1.556 | 158.20 ± 2.354 | 0.35 | 161.80 ± 1.218 | 161.67 ± 1.772 | 0.95 |
| (ICA PcoA) /L | 54.57 ± 1.888 | 57.00 ± 1.483 | 0.37 | 56.20 ± 1.679 | 55.56 ± 1.701 | 0.79 |
| (ICA PcoA) /R | 53.14 ± 1.580 | 54.00 ± 1.761 | 0.73 | 54.60 ± 1.720 | 58.56 ± 1.482 | 0.10 |
| PCA /L PCA /R | 123.86 ± 5.680 | 132.00 ± 4.572 | 0.32 | 114.00 ± 3.609 | 122.56 ± 5.580 | 0.21 |
| $R_{1,2}$ /M | 148.70 ± 30.776 | 133.27 ± 45.956 | 0.78 | 140.12 ± 17.534 | 144.77 ± 32.056 | 0.90 |
| $R_{3,4}$ /L | 55.63 ± 8.187 | 65.46 ± 12.108 | 0.50 | 53.96 ± 6.932 | 65.84 ± 6.176 | 0.22 |
| $R_{5,6}$ /R | 58.94 ± 5.484 | 99.95 ± 41.737 | 0.27 | 60.95 ± 5.893 | 99.24 ± 14.945 | **0.02** |
| $R_{7,8}$ /L | 31.14 ± 8.216 | 36.24 ± 13.095 | 0.74 | 23.96 ± 2.383 | 27.80 ± 3.051 | 0.33 |
| $R_{9,10}$ /R | 34.45 ± 6.183 | 49.07 ± 25.229 | 0.52 | 36.74 ± 4.468 | 37.67 ± 5.507 | 0.90 |
| $R_{11,12}$/L | 126.57 ± 27.091 | 174.60 ± 77.216 | 0.52 | 113.55 ± 21.885 | 156.14 ± 33.838 | 0.30 |
| $R_{13,14}$ /R | 118.52 ± 34.041 | 126.98 ± 58.833 | 0.90 | 149.61 ± 34.765 | 123.00 ± 22.381 | 0.54 |
| $R_{15,16}$ /R | 123.61 ± 26.765 | 175.01 ± 101.067 | 0.58 | 194.36 ± 55.111 | 106.44 ± 22.940 | 0.18 |
| $R_{17,18}$ /L | 119.37 ± 25.966 | 182.82 ± 59.428 | 0.30 | 92.98 ± 19.780 | 94.71 ± 19.822 | 0.95 |
| $R_{19,20}$ /R | 162.33 ± 29.572 | 157.76 ± 20.467 | 0.91 | 208.65 ± 56.701 | 249.12 ± 57.252 | 0.62 |
| $R_{20,21}$ /L | 118.32 ± 24.024 | 131.96 ± 30.657 | 0.73 | 209.36 ± 35.617 | 176.04 ± 33.737 | 0.51 |
| $S_{1}$ /M | 0.07 ± 0.009 | 0.07 ± 0.010 | 0.71 | 0.06 ± 0.006 | 0.11 ± 0.016 | **0.01** |
| $S_{2}$ /M | 0.06 ± 0.008 | 0.07 ± 0.008 | 0.62 | 0.06 ± 0.009 | 0.07 ± 0.012 | 0.45 |
| $S_{3}$ /L | 0.10 ± 0.004 | 0.09 ± 0.009 | 0.34 | 0.12 ± 0.009 | 0.09 ± 0.005 | **0.03** |
| $S_{4}$ /L | 0.12 ± 0.010 | 0.09 ± 0.014 | 0.14 | 0.11 ± 0.010 | 0.10 ± 0.006 | 0.16 |
| $S_{5}$ /R | 0.10 ± 0.008 | 0.09 ± 0.015 | 0.58 | 0.10 ± 0.009 | 0.08 ± 0.006 | 0.09 |
| $S_{6}$ /R | 0.08 ± 0.004 | 0.07 ± 0.006 | 0.22 | 0.10 ± 0.008 | 0.07 ± 0.004 | **0.01** |
| $S_{7}$ /L | 0.06 ± 0.006 | 0.06 ± 0.010 | 0.84 | 0.07 ± 0.007 | 0.07 ± 0.007 | 0.70 |
| $S_{8}$ /L | 0.07 ± 0.008 | 0.07 ± 0.010 | 0.92 | 0.07 ± 0.005 | 0.08 ± 0.005 | 0.76 |
| $S_{9}$ /R | 0.06 ± 0.007 | 0.06 ± 0.011 | 0.96 | 0.05 ± 0.003 | 0.06 ± 0.008 | 0.64 |
| $S_{10}$ /R | 0.07 ± 0.007 | 0.06 ± 0.009 | 0.62 | 0.06 ± 0.003 | 0.07 ± 0.007 | 0.86 |
| $S_{11}$ /L | 0.05 ± 0.007 | 0.04 ± 0.007 | 0.59 | 0.05 ± 0.005 | 0.04 ± 0.006 | 0.34 |
| $S_{12}$ /L | 0.05 ± 0.007 | 0.06 ± 0.011 | 0.58 | 0.05 ± 0.007 | 0.05 ± 0.009 | 0.83 |
| $S_{13}$ /R | 0.08 ± 0.006 | 0.07 ± 0.012 | 0.68 | 0.05 ± 0.006 | 0.04 ± 0.005 | 0.25 |
| $S_{14}$ /R | 0.03 ± 0.004 | 0.03 ± 0.005 | 0.87 | 0.04 ± 0.006 | 0.04 ± 0.003 | 0.69 |
| $S_{15}$ /R | 0.05 ± 0.005 | 0.05 ± 0.015 | 0.93 | 0.04 ± 0.006 | 0.05 ± 0.006 | 0.20 |
| $S_{16}$ /R | 0.05 ± 0.006 | 0.05 ± 0.010 | 0.56 | 0.05 ± 0.004 | 0.05 ± 0.004 | 0.92 |
| $S_{17}$ /L | 0.06 ± 0.003 | 0.06 ± 0.008 | 0.92 | 0.04 ± 0.004 | 0.05 ± 0.008 | 0.27 |
| $S_{18}$ /L | 0.05 ± 0.007 | 0.06 ± 0.011 | 0.47 | 0.04 ± 0.005 | 0.05 ± 0.005 | 0.29 |
| $S_{19}$ /R | 0.05 ± 0.010 | 0.06 ± 0.007 | 0.48 | 0.04 ± 0.004 | 0.04 ± 0.006 | 0.90 |
| $S_{20}$ /L | 0.06 ± 0.012 | 0.06 ± 0.008 | 0.94 | 0.06 ± 0.008 | 0.06 ± 0.005 | 0.93 |
| $S_{21}$ /L | 0.06 ± 0.014 | 0.05 ± 0.003 | 0.32 | 0.04 ± 0.006 | 0.04 ± 0.005 | 0.38 |
| $S_{22}$ /R | 0.04 ± 0.006 | 0.04 ± 0.003 | 0.89 | 0.04 ± 0.004 | 0.05 ± 0.006 | 0.26 |
| $Q_{1,2}$ /M | 5.72 ± 0.001 | 6.51 ± 0.001 | 0.43 | 5.53 ± 0.001 | 5.23 ± 0.0004 | 0.61 |
| $Q_{3,4}$ /L | 9.21 ± 0.001 | 6.46 ± 0.002 | 0.27 | 9.14 ± 0.001 | 9.42 ± 0.002 | 0.90 |
| $Q_{5,6}$ /R | 7.35 ± 0.002 | 7.53 ± 0.002 | 0.94 | 7.64 ± 0.001 | 5.12 ± 0.001 | 0.14 |
| $Q_{7,8}$ /L | 6.70 ± 0.002 | 3.92 ± 0.001 | 0.29 | 4.27 ± 0.001 | 4.62 ± 0.001 | 0.75 |
| $Q_{9,10}$ /R | 4.06 ± 0.001 | 4.05 ± 0.001 | 1.00 | 3.12 ± 0.001 | 3.26 ± 0.0004 | 0.85 |
| $Q_{11,12}$/L | 3.04 ± 0.001 | 4.31 ± 0.002 | 0.55 | 3.42 ± 0.001 | 2.38 ± 0.001 | 0.29 |
| $Q_{13,14}$ /R | 3.51 ± 0.001 | 4.29 ± 0.001 | 0.64 | 2.95 ± 0.001 | 4.18 ± 0.001 | 0.34 |
| $Q_{15,16}$ /R | 3.56 ± 0.001 | 3.76 ± 0.001 | 0.89 | 4.52 ± 0.001 | 2.45 ± 0.0004 | **0.05** |
| $Q_{17,18}$ /L | 5.17 ± 0.002 | 4.56 ± 0.001 | 0.79 | 4.88 ± 0.001 | 4.79 ± 0.001 | 0.95 |
| $Q_{19,20}$ /R | 3.03 ± 0.001 | 2.28 ± 0.0001 | 0.48 | 1.59 ± 0.001 | 1.62 ± 0.0002 | 0.94 |
| $Q_{20,21}$ /L | 4.43 ± 0.002 | 2.10 ± 0.0001 | 0.30 | 2.50 ± 0.001 | 2.89 ± 0.001 | 0.63 |
| $v_{1}^{max}$ /M | 14.38 ± 1.851 | 14.96 ± 2.303 | 0.85 | 14.22 ± 0.649 | 9.59 ± 1.038 | **0.001** |
| $v_{2}^{max}$ /M | 16.88 ± 1.854 | 18.08 ± 2.929 | 0.72 | 19.54 ± 1.559 | 17.80 ± 2.044 | 0.50 |
| $v_{3}^{max}$ /L | 15.10 ± 1.854 | 11.96 ± 2.475 | 0.32 | 12.47 ± 1.134 | 15.24 ± 1.898 | 0.22 |
| $v_{4}^{max}$ /L | 16.75 ± 2.775 | 14.05 ± 4.131 | 0.58 | 14.41 ± 1.598 | 16.33 ± 2.498 | 0.52 |
| $v_{5}^{max}$ /R | 12.96 ± 2.592 | 14.01 ± 2.104 | 0.77 | 13.81 ± 2.467 | 11.45 ± 2.176 | 0.49 |
| $v_{6}^{max}$ /R | 14.11 ± 1.911 | 14.18 ± 2.125 | 0.98 | 12.28 ± 1.610 | 11.96 ± 1.869 | 0.90 |
| $v_{7}^{max}$ /L | 17.08 ± 4.171 | 12.09 ± 2.109 | 0.37 | 10.16 ± 1.150 | 10.90 ± 0.953 | 0.63 |
| $v_{8}^{max}$ /L | 15.55 ± 3.107 | 11.74 ± 1.775 | 0.36 | 9.80 ± 0.921 | 11.03 ± 1.410 | 0.47 |
| $v_{9}^{max}$ /R | 12.98 ± 3.544 | 12.89 ± 2.414 | 0.99 | 9.88 ± 1.670 | 10.43 ± 1.328 | 0.80 |
| $v_{10}^{max}$ /R | 11.82 ± 3.169 | 12.24 ± 1.949 | 0.92 | 8.62 ± 1.398 | 8.75 ± 0.874 | 0.94 |
| $v_{11}^{max}$ /L | 15.42 ± 4.700 | 21.20 ± 9.503 | 0.56 | 14.08 ± 3.603 | 10.01 ± 1.975 | 0.35 |
| $v_{12}^{max}$ /L | 12.88 ± 4.050 | 13.60 ± 7.265 | 0.93 | 15.01 ± 4.549 | 8.63 ± 1.988 | 0.23 |
| $v_{13}^{max}$ /R | 11.01 ± 2.729 | 13.22 ± 3.464 | 0.62 | 9.04 ± 1.129 | 16.79 ± 3.880 | **0.06** |
| $v_{14}^{max}$ /R | 14.39 ± 2.817 | 21.55 ± 8.515 | 0.38 | 13.76 ± 2.980 | 18.23 ± 4.454 | 0.41 |
| $v_{15}^{max}$ /R | 16.82 ± 5.165 | 25.32 ± 14.930 | 0.55 | 24.77 ± 4.851 | 9.02 ± 1.396 | **0.01** |
| $v_{16}^{max}$ /R | 12.39 ± 3.089 | 17.46 ± 6.822 | 0.47 | 15.01 ± 2.456 | 8.84 ± 1.782 | **0.06** |
| $v_{17}^{max}$ /L | 14.37 ± 3.742 | 14.91 ± 3.472 | 0.92 | 18.81 ± 4.612 | 15.09 ± 2.918 | 0.52 |
| $v_{18}^{max}$/L | 27.56 ± 10.707 | 23.67 ± 4.646 | 0.78 | 21.62 ± 5.178 | 17.09 ± 2.930 | 0.47 |
| $v_{19}^{max}$ /R | 11.66 ± 2.903 | 7.45 ± 0.997 | 0.27 | 6.82 ± 1.747 | 8.09 ± 1.400 | 0.58 |
| $v_{20}^{max}$ /L | 10.85 ± 2.587 | 7.24 ± 1.227 | 0.29 | 7.17 ± 1.147 | 8.33 ± 1.106 | 0.48 |
| $v_{21}^{max}$ /L | 13.49 ± 2.791 | 7.93 ± 0.431 | 0.13 | 12.69 ± 3.173 | 7.93 ± 0.745 | 0.18 |
| $v_{22}^{max}$/R | 12.06 ± 1.326 | 8.13 ± 0.666 | **0.04** | 6.91 ± 1.600 | 10.71 ± 1.311 | 0.09 |

Values of the blood flow velocity at left and right Common cerebral artery (CCA) are shown in Table 4.

**Table 5: Blood flow velocity.** $[cm \cdot s^{-1}]$

|  | **Animal number** | $CCA_{L}$ | $CCA_{R}$ |
| --- | --- | --- | --- |
| **Group 1c** | 1 | 9.2 | 9.9 |
|  | 2 | 12.3 | 11.8 |
|  | 3 | 16 | 12.6 |
|  | 4 | 12.8 | 15.3 |
|  | 5 | 11.3 | 12.1 |
|  | 6 | 11 | 7.0 |
|  | 7 | 11.6 | 9.5 |
| **Group 1d** | 8 | 7.9 | 5.6 |
|  | 9 | 8.6 | 9.1 |
|  | 10 | 8.6 | 7.8 |
|  | 11 | 5.6 | 4.2 |
|  | 12 | 10.8 | 10.4 |
| **Group 2c** | 13 | 8.6 | 6.8 |
|  | 14 | 7.9 | 8.7 |
|  | 15 | 9.0 | 9.0 |
|  | 16 | 6.9 | 9.8 |
|  | 17 | 7.3 | 8.4 |
|  | 18 | 11.7 | 10.2 |
|  | 19 | 11.4 | 8.6 |
|  | 20 | 14 | 13.9 |
|  | 21 | 10.6 | 8.3 |
|  | 22 | 14.7 | 15.3 |
| **Group 2d** | 23 | 4.7 | 5 |
|  | 24 | 17.9 | 11.7 |
|  | 25 | 11 | 9.2 |
|  | 26 | 7.7 | 6.8 |
|  | 27 | 6.8 | 5.5 |
|  | 28 | 10.8 | 8.5 |
|  | 29 | 9.6 | 8.3 |
|  | 30 | 7.5 | 7.4 |
|  | 31 | 7.5 | 7.2 |
|  | 32 | 8.5 | 7.2 |
